# 2,2′,2′′-[(2,4,6-Trimeth­oxy­benzene-1,3,5-tri­yl)tris­(methyl­ene)]tris­(isoindole-1,3-dione)

**DOI:** 10.1107/S1600536813008428

**Published:** 2013-04-10

**Authors:** Jan-Ruven Rosien, Wilhelm Seichter, Monika Mazik

**Affiliations:** a Institut für Organische Chemie, Technische Universität Bergakademie Freiberg, Leipziger Strasse 29, D-09596 Freiberg/Sachsen, Germany

## Abstract

The title mol­ecule, C_36_H_27_N_3_O_9_, adopts an almost symmetric conformation in which the mean planes of the phthalimido units are inclined at dihedral angles of 81.1 (1), 85.3 (1) and 86.3 (1)° with respect to the plane of the central aromatic ring. The O atoms are involved in intra- and inter­molecular C—H⋯O hydrogen bonding. The crystal structure also features π–π arene inter­actions [minimum ring centroid separation = 3.683 (2) Å]. The present mode of non-covalent interactions leads to a three-dimensional supramolecular architecture.

## Related literature

For hydrogen bonds in the solid state, see: Desiraju (2002 ▶); Desiraju & Steiner (1999 ▶); Steiner (2002 ▶). For C—H⋯O hydrogen bonds in ketones carrying a terminal pyridine subunit, see: Mazik *et al.* (2001 ▶). For a review on acyclic receptors based on a benzene-derived core, see: Mazik (2009 ▶).
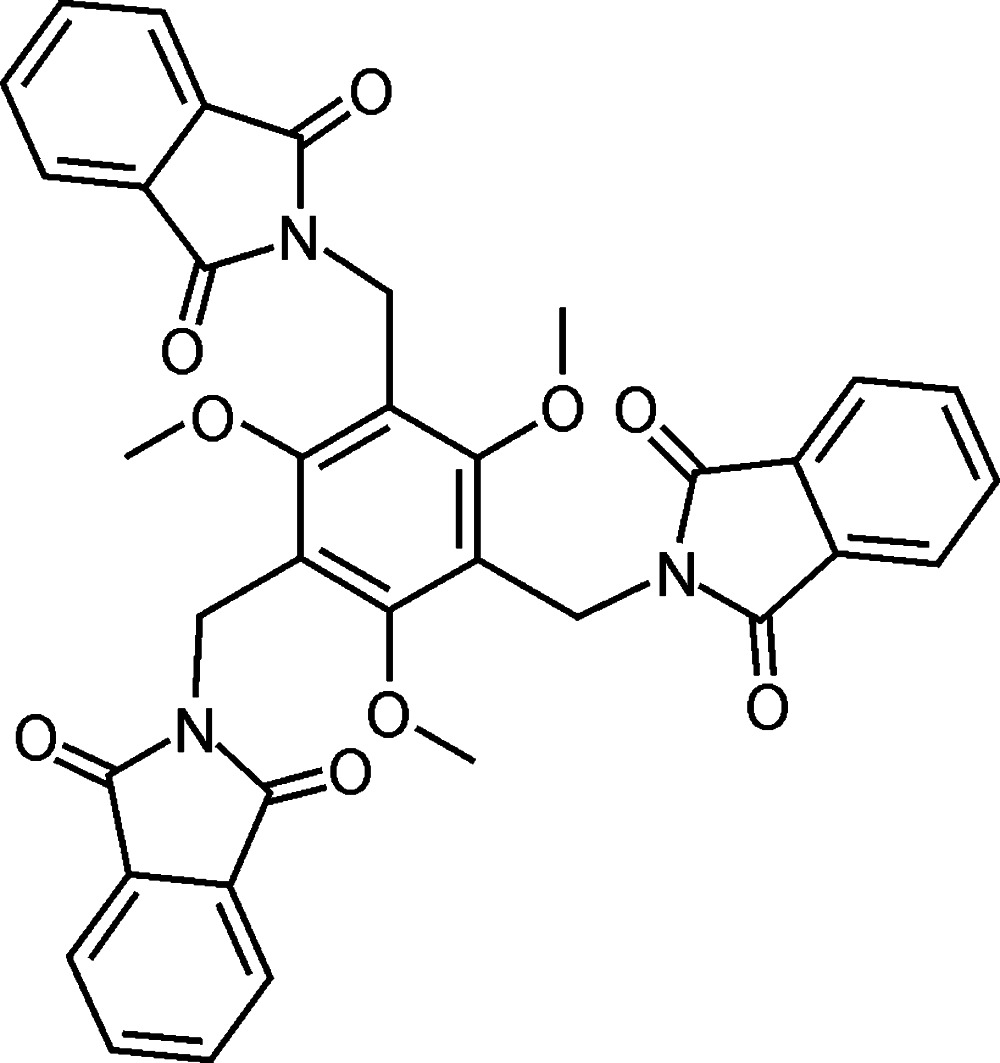


## Experimental

### 

#### Crystal data

C_36_H_27_N_3_O_9_
*M* *_r_* = 645.61
Triclinic, 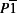

*a* = 10.2370 (3) Å
*b* = 10.3671 (3) Å
*c* = 14.6501 (4) Å
α = 79.804 (1)°
β = 79.512 (1)°
γ = 83.874 (1)°
*V* = 1500.20 (7) Å^3^
*Z* = 2
Mo *K*α radiation
μ = 0.10 mm^−1^
*T* = 100 K
0.51 × 0.50 × 0.28 mm

#### Data collection

Bruker APEXII CCD area-detector diffractometer
Absorption correction: multi-scan (*SADABS*; Bruker, 2008 ▶) *T*_min_ = 0.949, *T*_max_ = 0.971
29692 measured reflections
8026 independent reflections
6992 reflections with *I* > 2σ(*I*)
*R*_int_ = 0.021

#### Refinement

*R*[*F* ^2^ > 2σ(*F* ^2^)] = 0.039
*wR*(*F* ^2^) = 0.100
*S* = 0.96
8026 reflections
436 parameters
H-atom parameters constrained
Δρ_max_ = 0.46 e Å^−3^
Δρ_min_ = −0.35 e Å^−3^

### 

Data collection: *APEX2* (Bruker, 2008 ▶); cell refinement: *SAINT* (Bruker, 2008 ▶); data reduction: *SAINT*; program(s) used to solve structure: *SHELXS97* (Sheldrick, 2008 ▶); program(s) used to refine structure: *SHELXL97* (Sheldrick, 2008 ▶); molecular graphics: *ORTEP-3 for Windows* (Farrugia, 2012 ▶); software used to prepare material for publication: *SHELXTL* (Sheldrick, 2008 ▶).

## Supplementary Material

Crystal structure: contains datablock(s) I, global. DOI: 10.1107/S1600536813008428/zq2198sup1.cif

Structure factors: contains datablock(s) I. DOI: 10.1107/S1600536813008428/zq2198Isup2.hkl

Supplementary material file. DOI: 10.1107/S1600536813008428/zq2198Isup3.cml

Additional supplementary materials:  crystallographic information; 3D view; checkCIF report

## Figures and Tables

**Table 1:** Hydrogen-bond geometry (Å, °)

*D*—H⋯*A*	*D*—H	H⋯*A*	*D*⋯*A*	*D*—H⋯*A*
C7—H7*A*⋯O8^i^	0.98	2.47	3.2068 (15)	132
C8—H8*B*⋯O5^i^	0.98	2.34	3.2920 (15)	165
C9—H9*A*⋯O4^ii^	0.98	2.56	3.4742 (15)	156
C9—H9*C*⋯O9	0.98	2.38	3.2684 (19)	151
C10—H10*A*⋯O4	0.99	2.54	2.9144 (14)	102
C10—H10*B*⋯O3	0.99	2.32	2.8017 (15)	109
C19—H19*A*⋯O7	0.99	2.54	2.9200 (15)	103
C19—H19*B*⋯O2	0.99	2.36	2.8457 (14)	109
C23—H23⋯O1^iii^	0.95	2.45	3.3231 (16)	153
C28—H28*A*⋯O8	0.99	2.52	2.9187 (16)	103
C28—H28*B*⋯O3	0.99	2.34	2.8289 (16)	109
C32—H32⋯O2^iv^	0.95	2.56	3.2917 (16)	134
C34—H34⋯O7^v^	0.95	2.46	3.4066 (15)	172

Symmetry codes: (i) 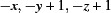; (ii) 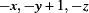; (iii) 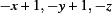; (iv) 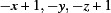; (v) 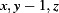.

**Fig. 1.**
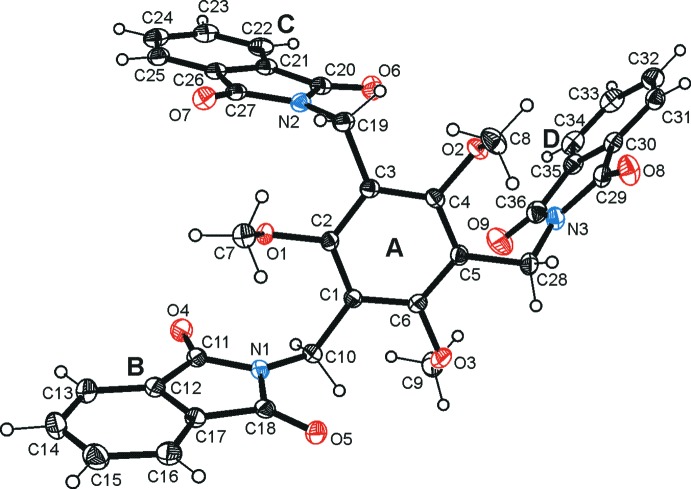
Perspective view of the title compound including the atom numbering. Anisotropic displacement parameters for non-hydrogen atoms are drawn at a 50% probability level.

**Fig. 2.**
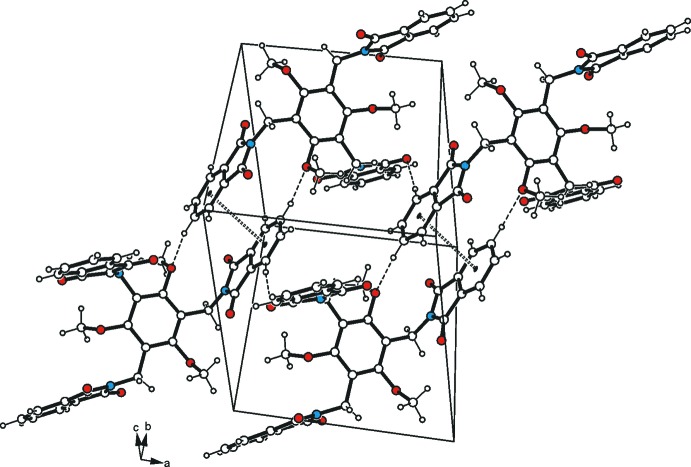
Packing diagram of the title compound. Hydrogen bonds are displayed as broken lines, while broken double lines represent π···π arene interactions.

## supplementary crystallographic information

### Comment

Our interest in the title compound, C_36_H_27_N_3_O_9_, arises from its use as an important precursor in the synthesis of artificial receptors based on trimethoxybenzene-derived core (for a review on acyclic carbohydrate receptors containing a trimethyl- or triethylbenzene scaffold, see: Mazik, 2009). The title compound crystallizes in the space group *P*-1 with one molecule in the asymmetric part of the unit cell. The interplanar angles between the phthalimido residues are 6.67 (4) (B/C), 48.86 (3) (B/D) and 53.23 (3)° (C/D). According to the three-dimensional arrangement of substituents around the benzene ring the present conformational isomer can be named 1-up, 3,5-down tris(phthalimidomethyl), 2,4-up, 6-down- trimethoxybenzene. The molecular conformation is stabilized by seven C—H···O hydrogen bonds (Desiraju, 2002; Desiraju & Steiner, 1999; Mazik *et al.*, 2001; Steiner, 2002) with phthalimido O atoms O(4), O(7), O(8), O(9) and the ether O atoms O(2), O(3) acting as acceptors [C—H···O_phthal_ 2.38 - 2.54 Å, C—H···O_ether_ 2.32 - 2.36 Å). This high degree of intramolecular hydrogen bonding may explain the slight twist of the benzene ring with maximum atomic distances from the best plane being -0.044 (1) and 0.027 (1) Å for C(4) and C(3). The packing structure is stabilized by intermolecular C—H···O bonding [C—H···O 2.34 - 2.54 Å, 132.1 - 172.1 °] as well as face-to-face arene interactions (centroid···centroid distances 3.683 (2), 3.693 (2) Å].

### Experimental

A mixture of 1,3,5-tris(bromomethyl)-2,4,6-trimethoxybenzene (5.4 g, 12.0 mmol) and potassium phthalimide (10.0 g, 54.0 mmol) suspended in dry dimethyl sulfoxide (150 ml) was stirred at 120 °C for 8 h. After the mixture was cooled to room temperature, water (300 ml) was added and the formed precipitate was filtered and washed with water (400 ml). Then the precipitate was suspended in water (150 ml), and the suspension was extracted with chloroform (3 *x* 100 ml). The combined organic layers were washed with brine (100 ml) and water (100 ml), dried over magnesium sulfate and concentrated *in vacuo*. The desired product was obtained as a white solid after flash chromatography (SiO_2_, toluene/ethyl acetate 2:1 *v/v*, *R_f_* = 0.37) in 46% yield (3.5 g, 5.5 mmol).

Analysis data: m.p. = 235 °C; ^1^H-NMR (400 MHz, CDCl_3_) δ 7.68 (m, 6H), 7.60 (m, 6H), 4.87 (s, 6H), 3.89 (s, 9H) p.p.m.; ^13^C-NMR (100 MHz, CDCl_3_) δ 167.83, 159.07, 133.55, 132.03, 122.95, 119.12, 62.21, 32.80 p.p.m..

Suitable crystals of the title compound for X-ray analysis were obtained by slow evaporation of a CHCl_3_ solution.

### Crystal data

**Table:**

C_36_H_27_N_3_O_9_	*Z* = 2
*M**_r_* = 645.61	*F*(000) = 672
Triclinic, *P*1	*D*_x_ = 1.429 Mg m^−^^3^
Hall symbol: -P 1	Mo *K*α radiation, λ = 0.71073 Å
*a* = 10.2370 (3) Å	Cell parameters from 9907 reflections
*b* = 10.3671 (3) Å	θ = 2.3–29.2°
*c* = 14.6501 (4) Å	µ = 0.10 mm^−^^1^
α = 79.804 (1)°	*T* = 100 K
β = 79.512 (1)°	Irregular, colourless
γ = 83.874 (1)°	0.51 × 0.50 × 0.28 mm
*V* = 1500.20 (7) Å^3^	

### Data collection

**Table:**

Bruker APEXII CCD area-detector diffractometer	8026 independent reflections
Radiation source: fine-focus sealed tube	6992 reflections with *I* > 2σ(*I*)
Graphite monochromator	*R*_int_ = 0.021
phi and ω scans	θ_max_ = 29.1°, θ_min_ = 1.4°
Absorption correction: multi-scan (*SADABS*; Bruker, 2008)	*h* = −14→13
*T*_min_ = 0.949, *T*_max_ = 0.971	*k* = −14→14
29692 measured reflections	*l* = −20→20

### Refinement

**Table:**

Refinement on *F*^2^	Primary atom site location: structure-invariant direct methods
Least-squares matrix: full	Secondary atom site location: difference Fourier map
*R*[*F*^2^ > 2σ(*F*^2^)] = 0.039	Hydrogen site location: inferred from neighbouring sites
*wR*(*F*^2^) = 0.100	H-atom parameters constrained
*S* = 0.96	*w* = 1/[σ^2^(*F*_o_^2^) + (0.0441*P*)^2^ + 0.958*P*] where *P* = (*F*_o_^2^ + 2*F*_c_^2^)/3
8026 reflections	(Δ/σ)_max_ < 0.001
436 parameters	Δρ_max_ = 0.46 e Å^−^^3^
0 restraints	Δρ_min_ = −0.35 e Å^−^^3^

### Special details

**Table:**

Geometry. All e.s.d.'s (except the e.s.d. in the dihedral angle between two l.s. planes) are estimated using the full covariance matrix. The cell e.s.d.'s are taken into account individually in the estimation of e.s.d.'s in distances, angles and torsion angles; correlations between e.s.d.'s in cell parameters are only used when they are defined by crystal symmetry. An approximate (isotropic) treatment of cell e.s.d.'s is used for estimating e.s.d.'s involving l.s. planes.
Refinement. Refinement of *F*^2^ against ALL reflections. The weighted *R*-factor *wR* and goodness of fit *S* are based on *F*^2^, conventional *R*-factors *R* are based on *F*, with *F* set to zero for negative *F*^2^. The threshold expression of *F*^2^ > σ(*F*^2^) is used only for calculating *R*-factors(gt) *etc*. and is not relevant to the choice of reflections for refinement. *R*-factors based on *F*^2^ are statistically about twice as large as those based on *F*, and *R*- factors based on ALL data will be even larger.

### Fractional atomic coordinates and isotropic or equivalent isotropic displacement parameters (Å^2^)

**Table:**

	*x*	*y*	*z*	*U*_iso_*/*U*_eq_	
O1	0.12796 (8)	0.66212 (7)	0.18783 (5)	0.01633 (15)	
O2	0.22988 (8)	0.36078 (8)	0.45741 (5)	0.01958 (17)	
O3	−0.12369 (9)	0.28557 (9)	0.30721 (6)	0.02355 (18)	
O4	0.00470 (9)	0.69783 (9)	0.00364 (6)	0.02422 (18)	
O5	−0.30723 (8)	0.65590 (9)	0.27100 (6)	0.02150 (17)	
O6	0.48284 (9)	0.35909 (9)	0.24204 (6)	0.02684 (19)	
O7	0.38511 (8)	0.80269 (8)	0.17348 (6)	0.02226 (17)	
O8	0.15450 (10)	0.11530 (9)	0.61971 (6)	0.0286 (2)	
O9	0.16627 (11)	0.05026 (11)	0.31774 (7)	0.0358 (2)	
N1	−0.13167 (9)	0.64776 (9)	0.14771 (6)	0.01741 (18)	
N2	0.40392 (9)	0.57826 (9)	0.22568 (7)	0.01757 (18)	
N3	0.13324 (10)	0.10612 (10)	0.46635 (7)	0.0210 (2)	
C1	0.00047 (10)	0.47455 (10)	0.24429 (7)	0.01530 (19)	
C2	0.10435 (10)	0.54768 (10)	0.25067 (7)	0.01478 (19)	
C3	0.18863 (10)	0.50669 (11)	0.31740 (7)	0.0156 (2)	
C4	0.15679 (11)	0.39756 (11)	0.38509 (7)	0.0164 (2)	
C5	0.05402 (11)	0.32162 (11)	0.38223 (8)	0.0177 (2)	
C6	−0.02044 (11)	0.35899 (11)	0.30973 (8)	0.0172 (2)	
C7	0.08112 (12)	0.77913 (11)	0.22889 (8)	0.0208 (2)	
H7A	−0.0131	0.7745	0.2571	0.031*	
H7B	0.0913	0.8568	0.1799	0.031*	
H7C	0.1334	0.7854	0.2775	0.031*	
C8	0.18680 (13)	0.43689 (14)	0.53196 (8)	0.0265 (3)	
H8A	0.2009	0.5296	0.5079	0.040*	
H8B	0.2382	0.4053	0.5829	0.040*	
H8C	0.0919	0.4277	0.5560	0.040*	
C9	−0.09626 (15)	0.20079 (13)	0.23660 (9)	0.0295 (3)	
H9A	−0.0609	0.2516	0.1757	0.044*	
H9B	−0.1787	0.1641	0.2319	0.044*	
H9C	−0.0305	0.1291	0.2542	0.044*	
C10	−0.08810 (11)	0.50962 (11)	0.16956 (8)	0.0177 (2)	
H10A	−0.0395	0.4804	0.1110	0.021*	
H10B	−0.1681	0.4592	0.1903	0.021*	
C11	−0.08304 (11)	0.72973 (11)	0.06444 (8)	0.0187 (2)	
C12	−0.16249 (11)	0.85805 (11)	0.06850 (8)	0.0191 (2)	
C13	−0.15858 (13)	0.97272 (12)	0.00389 (9)	0.0247 (2)	
H13	−0.0977	0.9794	−0.0537	0.030*	
C14	−0.24767 (14)	1.07832 (13)	0.02669 (9)	0.0275 (3)	
H14	−0.2475	1.1586	−0.0162	0.033*	
C15	−0.33658 (13)	1.06813 (12)	0.11105 (10)	0.0270 (3)	
H15	−0.3945	1.1423	0.1255	0.032*	
C16	−0.34221 (12)	0.95084 (12)	0.17486 (9)	0.0225 (2)	
H16	−0.4042	0.9428	0.2319	0.027*	
C17	−0.25397 (11)	0.84723 (11)	0.15155 (8)	0.0181 (2)	
C18	−0.23948 (11)	0.70940 (11)	0.20107 (7)	0.0172 (2)	
C19	0.31307 (11)	0.57446 (12)	0.31515 (8)	0.0191 (2)	
H19A	0.2872	0.6655	0.3271	0.023*	
H19B	0.3601	0.5280	0.3663	0.023*	
C20	0.48423 (11)	0.46919 (12)	0.19829 (8)	0.0194 (2)	
C21	0.56744 (11)	0.51949 (12)	0.10683 (8)	0.0187 (2)	
C22	0.66502 (12)	0.45525 (13)	0.04912 (9)	0.0234 (2)	
H22	0.6892	0.3640	0.0648	0.028*	
C23	0.72661 (12)	0.52973 (14)	−0.03315 (9)	0.0268 (3)	
H23	0.7941	0.4883	−0.0743	0.032*	
C24	0.69162 (12)	0.66280 (14)	−0.05616 (8)	0.0263 (3)	
H24	0.7341	0.7105	−0.1134	0.032*	
C25	0.59480 (12)	0.72803 (13)	0.00356 (8)	0.0218 (2)	
H25	0.5716	0.8196	−0.0111	0.026*	
C26	0.53433 (11)	0.65336 (11)	0.08482 (8)	0.0179 (2)	
C27	0.43267 (11)	0.69367 (11)	0.16284 (8)	0.0176 (2)	
C28	0.02137 (12)	0.20410 (12)	0.45709 (9)	0.0229 (2)	
H28A	−0.0095	0.2347	0.5183	0.028*	
H28B	−0.0529	0.1622	0.4421	0.028*	
C29	0.19020 (12)	0.07027 (11)	0.54809 (8)	0.0200 (2)	
C30	0.29838 (11)	−0.03345 (11)	0.52684 (8)	0.0189 (2)	
C31	0.38234 (12)	−0.10727 (11)	0.58341 (8)	0.0210 (2)	
H31	0.3772	−0.0964	0.6471	0.025*	
C32	0.47546 (13)	−0.19880 (13)	0.54295 (10)	0.0269 (3)	
H32	0.5346	−0.2516	0.5799	0.032*	
C33	0.48304 (13)	−0.21396 (12)	0.44909 (10)	0.0279 (3)	
H33	0.5482	−0.2755	0.4229	0.033*	
C34	0.39607 (13)	−0.13983 (12)	0.39372 (9)	0.0258 (3)	
H34	0.4000	−0.1503	0.3301	0.031*	
C35	0.30401 (12)	−0.05075 (11)	0.43437 (8)	0.0204 (2)	
C36	0.19708 (13)	0.03783 (12)	0.39463 (8)	0.0230 (2)	

### Atomic displacement parameters (Å^2^)

**Table:**

	*U*^11^	*U*^22^	*U*^33^	*U*^12^	*U*^13^	*U*^23^
O1	0.0186 (4)	0.0158 (4)	0.0135 (3)	−0.0023 (3)	−0.0002 (3)	−0.0014 (3)
O2	0.0177 (4)	0.0257 (4)	0.0146 (3)	0.0032 (3)	−0.0047 (3)	−0.0021 (3)
O3	0.0212 (4)	0.0260 (4)	0.0239 (4)	−0.0106 (3)	−0.0042 (3)	0.0005 (3)
O4	0.0219 (4)	0.0305 (5)	0.0179 (4)	−0.0017 (3)	0.0002 (3)	−0.0014 (3)
O5	0.0215 (4)	0.0266 (4)	0.0153 (4)	−0.0039 (3)	−0.0015 (3)	−0.0006 (3)
O6	0.0302 (5)	0.0228 (4)	0.0264 (4)	−0.0015 (4)	−0.0057 (4)	−0.0001 (3)
O7	0.0208 (4)	0.0216 (4)	0.0243 (4)	−0.0010 (3)	−0.0020 (3)	−0.0056 (3)
O8	0.0334 (5)	0.0295 (5)	0.0180 (4)	0.0082 (4)	0.0013 (3)	−0.0033 (3)
O9	0.0465 (6)	0.0388 (6)	0.0265 (5)	−0.0001 (5)	−0.0157 (4)	−0.0095 (4)
N1	0.0163 (4)	0.0197 (4)	0.0152 (4)	−0.0014 (3)	−0.0030 (3)	0.0003 (3)
N2	0.0150 (4)	0.0211 (5)	0.0163 (4)	−0.0027 (3)	−0.0009 (3)	−0.0031 (3)
N3	0.0231 (5)	0.0189 (5)	0.0190 (4)	0.0004 (4)	−0.0030 (4)	0.0004 (4)
C1	0.0141 (5)	0.0177 (5)	0.0134 (4)	−0.0003 (4)	−0.0016 (4)	−0.0020 (4)
C2	0.0145 (5)	0.0166 (5)	0.0119 (4)	−0.0005 (4)	0.0007 (3)	−0.0021 (4)
C3	0.0139 (5)	0.0193 (5)	0.0136 (4)	−0.0013 (4)	−0.0007 (4)	−0.0039 (4)
C4	0.0144 (5)	0.0203 (5)	0.0133 (4)	0.0024 (4)	−0.0018 (4)	−0.0022 (4)
C5	0.0163 (5)	0.0181 (5)	0.0162 (5)	0.0002 (4)	−0.0004 (4)	0.0005 (4)
C6	0.0149 (5)	0.0191 (5)	0.0169 (5)	−0.0031 (4)	−0.0010 (4)	−0.0017 (4)
C7	0.0221 (5)	0.0191 (5)	0.0209 (5)	0.0011 (4)	−0.0021 (4)	−0.0052 (4)
C8	0.0266 (6)	0.0377 (7)	0.0148 (5)	0.0056 (5)	−0.0050 (4)	−0.0064 (5)
C9	0.0396 (7)	0.0272 (6)	0.0254 (6)	−0.0136 (5)	−0.0121 (5)	−0.0009 (5)
C10	0.0174 (5)	0.0190 (5)	0.0171 (5)	−0.0017 (4)	−0.0054 (4)	−0.0012 (4)
C11	0.0176 (5)	0.0227 (5)	0.0157 (5)	−0.0041 (4)	−0.0044 (4)	0.0001 (4)
C12	0.0182 (5)	0.0217 (5)	0.0179 (5)	−0.0037 (4)	−0.0053 (4)	−0.0006 (4)
C13	0.0252 (6)	0.0257 (6)	0.0217 (5)	−0.0053 (5)	−0.0051 (4)	0.0031 (4)
C14	0.0316 (7)	0.0212 (6)	0.0290 (6)	−0.0029 (5)	−0.0104 (5)	0.0043 (5)
C15	0.0269 (6)	0.0213 (6)	0.0340 (7)	0.0006 (5)	−0.0102 (5)	−0.0040 (5)
C16	0.0214 (5)	0.0237 (6)	0.0237 (5)	−0.0023 (4)	−0.0051 (4)	−0.0051 (4)
C17	0.0177 (5)	0.0200 (5)	0.0177 (5)	−0.0036 (4)	−0.0062 (4)	−0.0010 (4)
C18	0.0164 (5)	0.0210 (5)	0.0153 (5)	−0.0024 (4)	−0.0053 (4)	−0.0026 (4)
C19	0.0168 (5)	0.0267 (6)	0.0144 (5)	−0.0050 (4)	−0.0012 (4)	−0.0043 (4)
C20	0.0164 (5)	0.0237 (5)	0.0197 (5)	−0.0021 (4)	−0.0054 (4)	−0.0052 (4)
C21	0.0152 (5)	0.0245 (5)	0.0184 (5)	−0.0032 (4)	−0.0042 (4)	−0.0063 (4)
C22	0.0188 (5)	0.0291 (6)	0.0258 (6)	−0.0003 (4)	−0.0052 (4)	−0.0127 (5)
C23	0.0177 (5)	0.0432 (7)	0.0231 (6)	−0.0041 (5)	−0.0005 (4)	−0.0169 (5)
C24	0.0207 (6)	0.0422 (7)	0.0175 (5)	−0.0104 (5)	−0.0003 (4)	−0.0076 (5)
C25	0.0188 (5)	0.0289 (6)	0.0190 (5)	−0.0069 (4)	−0.0041 (4)	−0.0030 (4)
C26	0.0139 (5)	0.0244 (5)	0.0170 (5)	−0.0034 (4)	−0.0030 (4)	−0.0059 (4)
C27	0.0138 (5)	0.0229 (5)	0.0171 (5)	−0.0037 (4)	−0.0036 (4)	−0.0038 (4)
C28	0.0193 (5)	0.0224 (6)	0.0227 (5)	−0.0006 (4)	−0.0019 (4)	0.0057 (4)
C29	0.0208 (5)	0.0184 (5)	0.0178 (5)	−0.0009 (4)	0.0001 (4)	0.0010 (4)
C30	0.0187 (5)	0.0173 (5)	0.0193 (5)	−0.0026 (4)	0.0007 (4)	−0.0026 (4)
C31	0.0203 (5)	0.0207 (5)	0.0203 (5)	−0.0008 (4)	−0.0004 (4)	−0.0022 (4)
C32	0.0201 (6)	0.0252 (6)	0.0322 (6)	0.0021 (5)	−0.0015 (5)	−0.0016 (5)
C33	0.0246 (6)	0.0212 (6)	0.0364 (7)	−0.0008 (5)	0.0021 (5)	−0.0085 (5)
C34	0.0291 (6)	0.0236 (6)	0.0254 (6)	−0.0064 (5)	0.0026 (5)	−0.0104 (5)
C35	0.0220 (5)	0.0186 (5)	0.0209 (5)	−0.0051 (4)	−0.0012 (4)	−0.0041 (4)
C36	0.0270 (6)	0.0210 (5)	0.0219 (5)	−0.0054 (4)	−0.0042 (4)	−0.0034 (4)

### Geometric parameters (Å, º)

**Table:**

O1—C2	1.3810 (13)	C11—C12	1.4881 (16)
O1—C7	1.4465 (13)	C12—C13	1.3816 (16)
O2—C4	1.3829 (13)	C12—C17	1.3889 (16)
O2—C8	1.4372 (14)	C13—C14	1.3955 (19)
O3—C6	1.3761 (13)	C13—H13	0.9500
O3—C9	1.4439 (16)	C14—C15	1.3899 (19)
O4—C11	1.2080 (14)	C14—H14	0.9500
O5—C18	1.2085 (14)	C15—C16	1.3960 (18)
O6—C20	1.2063 (15)	C15—H15	0.9500
O7—C27	1.2067 (14)	C16—C17	1.3772 (16)
O8—C29	1.2042 (14)	C16—H16	0.9500
O9—C36	1.2060 (15)	C17—C18	1.4889 (16)
N1—C18	1.3964 (14)	C19—H19A	0.9900
N1—C11	1.4000 (14)	C19—H19B	0.9900
N1—C10	1.4511 (14)	C20—C21	1.4915 (16)
N2—C27	1.3972 (14)	C21—C22	1.3806 (16)
N2—C20	1.3991 (15)	C21—C26	1.3878 (16)
N2—C19	1.4596 (14)	C22—C23	1.3944 (18)
N3—C36	1.3953 (15)	C22—H22	0.9500
N3—C29	1.4009 (15)	C23—C24	1.385 (2)
N3—C28	1.4564 (15)	C23—H23	0.9500
C1—C2	1.3956 (15)	C24—C25	1.3985 (17)
C1—C6	1.4048 (15)	C24—H24	0.9500
C1—C10	1.5176 (15)	C25—C26	1.3811 (16)
C2—C3	1.4008 (15)	C25—H25	0.9500
C3—C4	1.3905 (15)	C26—C27	1.4858 (15)
C3—C19	1.5116 (15)	C28—H28A	0.9900
C4—C5	1.3902 (16)	C28—H28B	0.9900
C5—C6	1.3947 (15)	C29—C30	1.4894 (16)
C5—C28	1.5098 (15)	C30—C31	1.3804 (16)
C7—H7A	0.9800	C30—C35	1.3877 (16)
C7—H7B	0.9800	C31—C32	1.4004 (17)
C7—H7C	0.9800	C31—H31	0.9500
C8—H8A	0.9800	C32—C33	1.3987 (19)
C8—H8B	0.9800	C32—H32	0.9500
C8—H8C	0.9800	C33—C34	1.392 (2)
C9—H9A	0.9800	C33—H33	0.9500
C9—H9B	0.9800	C34—C35	1.3786 (16)
C9—H9C	0.9800	C34—H34	0.9500
C10—H10A	0.9900	C35—C36	1.4862 (17)
C10—H10B	0.9900		
			
C2—O1—C7	112.85 (8)	C17—C16—C15	117.15 (11)
C4—O2—C8	112.02 (9)	C17—C16—H16	121.4
C6—O3—C9	114.65 (9)	C15—C16—H16	121.4
C18—N1—C11	111.94 (9)	C16—C17—C12	121.89 (11)
C18—N1—C10	123.58 (9)	C16—C17—C18	130.24 (11)
C11—N1—C10	123.92 (9)	C12—C17—C18	107.80 (10)
C27—N2—C20	111.99 (9)	O5—C18—N1	124.86 (11)
C27—N2—C19	123.90 (9)	O5—C18—C17	129.13 (11)
C20—N2—C19	123.69 (10)	N1—C18—C17	105.96 (9)
C36—N3—C29	112.05 (10)	N2—C19—C3	112.88 (9)
C36—N3—C28	124.02 (10)	N2—C19—H19A	109.0
C29—N3—C28	123.93 (10)	C3—C19—H19A	109.0
O3—C6—C5	118.58 (10)	N2—C19—H19B	109.0
O3—C6—C1	119.46 (10)	C3—C19—H19B	109.0
C5—C6—C1	121.76 (10)	H19A—C19—H19B	107.8
C2—C1—C6	117.61 (10)	O6—C20—N2	125.21 (11)
C2—C1—C10	124.23 (9)	O6—C20—C21	129.24 (11)
C6—C1—C10	118.13 (9)	N2—C20—C21	105.56 (10)
O1—C2—C1	119.42 (9)	C22—C21—C26	121.27 (11)
O1—C2—C3	118.54 (9)	C22—C21—C20	130.47 (11)
C1—C2—C3	122.02 (10)	C26—C21—C20	108.25 (10)
C4—C3—C2	117.75 (10)	C21—C22—C23	117.28 (12)
C4—C3—C19	120.26 (10)	C21—C22—H22	121.4
C2—C3—C19	121.96 (10)	C23—C22—H22	121.4
O2—C4—C5	118.08 (10)	C24—C23—C22	121.47 (11)
O2—C4—C3	119.75 (10)	C24—C23—H23	119.3
C5—C4—C3	122.17 (10)	C22—C23—H23	119.3
C4—C5—C6	118.20 (10)	C23—C24—C25	121.02 (12)
C4—C5—C28	120.91 (10)	C23—C24—H24	119.5
C6—C5—C28	120.86 (10)	C25—C24—H24	119.5
O1—C7—H7A	109.5	C26—C25—C24	117.04 (12)
O1—C7—H7B	109.5	C26—C25—H25	121.5
H7A—C7—H7B	109.5	C24—C25—H25	121.5
O1—C7—H7C	109.5	C25—C26—C21	121.89 (11)
H7A—C7—H7C	109.5	C25—C26—C27	129.93 (11)
H7B—C7—H7C	109.5	C21—C26—C27	108.15 (10)
O2—C8—H8A	109.5	O7—C27—N2	125.44 (10)
O2—C8—H8B	109.5	O7—C27—C26	128.63 (11)
H8A—C8—H8B	109.5	N2—C27—C26	105.90 (9)
O2—C8—H8C	109.5	N3—C28—C5	113.73 (9)
H8A—C8—H8C	109.5	N3—C28—H28A	108.8
H8B—C8—H8C	109.5	C5—C28—H28A	108.8
O3—C9—H9A	109.5	N3—C28—H28B	108.8
O3—C9—H9B	109.5	C5—C28—H28B	108.8
H9A—C9—H9B	109.5	H28A—C28—H28B	107.7
O3—C9—H9C	109.5	O8—C29—N3	125.31 (11)
H9A—C9—H9C	109.5	O8—C29—C30	129.03 (11)
H9B—C9—H9C	109.5	N3—C29—C30	105.65 (9)
N1—C10—C1	115.94 (9)	C31—C30—C35	121.58 (11)
N1—C10—H10A	108.3	C31—C30—C29	130.32 (10)
C1—C10—H10A	108.3	C35—C30—C29	108.09 (10)
N1—C10—H10B	108.3	C30—C31—C32	117.13 (11)
C1—C10—H10B	108.3	C30—C31—H31	121.4
H10A—C10—H10B	107.4	C32—C31—H31	121.4
O4—C11—N1	125.15 (11)	C31—C32—C33	121.20 (12)
O4—C11—C12	129.32 (11)	C31—C32—H32	119.4
N1—C11—C12	105.53 (9)	C33—C32—H32	119.4
C13—C12—C17	121.30 (11)	C34—C33—C32	120.69 (12)
C13—C12—C11	130.13 (11)	C34—C33—H33	119.7
C17—C12—C11	108.52 (10)	C32—C33—H33	119.7
C12—C13—C14	117.29 (12)	C35—C34—C33	117.68 (12)
C12—C13—H13	121.4	C35—C34—H34	121.2
C14—C13—H13	121.4	C33—C34—H34	121.2
C15—C14—C13	121.17 (12)	C34—C35—C30	121.68 (12)
C15—C14—H14	119.4	C34—C35—C36	129.94 (11)
C13—C14—H14	119.4	C30—C35—C36	108.36 (10)
C16—C15—C14	121.15 (12)	O9—C36—N3	124.89 (12)
C16—C15—H15	119.4	O9—C36—C35	129.29 (12)
C14—C15—H15	119.4	N3—C36—C35	105.81 (10)
			
C9—O3—C6—C5	−104.74 (12)	C20—N2—C19—C3	−74.87 (13)
C9—O3—C6—C1	80.27 (13)	C4—C3—C19—N2	121.69 (11)
O3—C6—C1—C2	178.55 (9)	C2—C3—C19—N2	−56.16 (14)
C5—C6—C1—C2	3.73 (16)	C27—N2—C20—O6	177.12 (11)
O3—C6—C1—C10	−3.32 (15)	C19—N2—C20—O6	4.32 (18)
C5—C6—C1—C10	−178.14 (10)	C27—N2—C20—C21	−2.87 (12)
C7—O1—C2—C1	103.89 (11)	C19—N2—C20—C21	−175.67 (9)
C7—O1—C2—C3	−77.97 (12)	O6—C20—C21—C22	−1.0 (2)
C6—C1—C2—O1	−179.81 (9)	N2—C20—C21—C22	178.97 (11)
C10—C1—C2—O1	2.18 (15)	O6—C20—C21—C26	−179.56 (12)
C6—C1—C2—C3	2.11 (15)	N2—C20—C21—C26	0.44 (12)
C10—C1—C2—C3	−175.90 (10)	C26—C21—C22—C23	−1.46 (17)
O1—C2—C3—C4	174.67 (9)	C20—C21—C22—C23	−179.82 (11)
C1—C2—C3—C4	−7.24 (15)	C21—C22—C23—C24	0.08 (17)
O1—C2—C3—C19	−7.44 (15)	C22—C23—C24—C25	1.35 (18)
C1—C2—C3—C19	170.66 (10)	C23—C24—C25—C26	−1.35 (17)
C8—O2—C4—C5	−99.36 (12)	C24—C25—C26—C21	−0.03 (17)
C8—O2—C4—C3	81.54 (12)	C24—C25—C26—C27	177.75 (11)
C2—C3—C4—O2	−174.09 (9)	C22—C21—C26—C25	1.47 (17)
C19—C3—C4—O2	7.98 (15)	C20—C21—C26—C25	−179.84 (10)
C2—C3—C4—C5	6.85 (16)	C22—C21—C26—C27	−176.74 (10)
C19—C3—C4—C5	−171.08 (10)	C20—C21—C26—C27	1.95 (12)
O2—C4—C5—C6	179.58 (9)	C20—N2—C27—O7	−174.20 (11)
C3—C4—C5—C6	−1.34 (16)	C19—N2—C27—O7	−1.42 (17)
O2—C4—C5—C28	1.27 (15)	C20—N2—C27—C26	4.04 (12)
C3—C4—C5—C28	−179.66 (10)	C19—N2—C27—C26	176.82 (9)
O3—C6—C5—C4	−178.99 (10)	C25—C26—C27—O7	−3.5 (2)
C1—C6—C5—C4	−4.12 (16)	C21—C26—C27—O7	174.53 (11)
O3—C6—C5—C28	−0.68 (16)	C25—C26—C27—N2	178.36 (11)
C1—C6—C5—C28	174.19 (10)	C21—C26—C27—N2	−3.63 (12)
C18—N1—C10—C1	−81.93 (13)	C36—N3—C28—C5	−61.67 (15)
C11—N1—C10—C1	107.26 (12)	C29—N3—C28—C5	117.70 (12)
C2—C1—C10—N1	−42.09 (15)	C4—C5—C28—N3	−57.36 (15)
C6—C1—C10—N1	139.92 (10)	C6—C5—C28—N3	124.37 (12)
C18—N1—C11—O4	−175.72 (11)	C36—N3—C29—O8	179.36 (12)
C10—N1—C11—O4	−3.96 (18)	C28—N3—C29—O8	−0.08 (19)
C18—N1—C11—C12	3.69 (12)	C36—N3—C29—C30	−1.62 (13)
C10—N1—C11—C12	175.45 (9)	C28—N3—C29—C30	178.94 (10)
O4—C11—C12—C13	1.2 (2)	O8—C29—C30—C31	2.3 (2)
N1—C11—C12—C13	−178.18 (12)	N3—C29—C30—C31	−176.70 (12)
O4—C11—C12—C17	178.78 (12)	O8—C29—C30—C35	−178.89 (13)
N1—C11—C12—C17	−0.59 (12)	N3—C29—C30—C35	2.13 (12)
C17—C12—C13—C14	1.78 (18)	C35—C30—C31—C32	1.23 (17)
C11—C12—C13—C14	179.10 (12)	C29—C30—C31—C32	179.92 (12)
C12—C13—C14—C15	−0.01 (19)	C30—C31—C32—C33	0.27 (18)
C13—C14—C15—C16	−1.7 (2)	C31—C32—C33—C34	−1.2 (2)
C14—C15—C16—C17	1.49 (18)	C32—C33—C34—C35	0.67 (19)
C15—C16—C17—C12	0.28 (17)	C33—C34—C35—C30	0.82 (18)
C15—C16—C17—C18	−176.42 (11)	C33—C34—C35—C36	−177.85 (12)
C13—C12—C17—C16	−1.96 (18)	C31—C30—C35—C34	−1.83 (18)
C11—C12—C17—C16	−179.80 (10)	C29—C30—C35—C34	179.22 (11)
C13—C12—C17—C18	175.39 (10)	C31—C30—C35—C36	177.10 (11)
C11—C12—C17—C18	−2.45 (12)	C29—C30—C35—C36	−1.85 (13)
C11—N1—C18—O5	172.43 (10)	C29—N3—C36—O9	179.40 (12)
C10—N1—C18—O5	0.64 (17)	C28—N3—C36—O9	−1.2 (2)
C11—N1—C18—C17	−5.16 (12)	C29—N3—C36—C35	0.52 (13)
C10—N1—C18—C17	−176.95 (9)	C28—N3—C36—C35	179.97 (10)
C16—C17—C18—O5	4.2 (2)	C34—C35—C36—O9	0.9 (2)
C12—C17—C18—O5	−172.84 (11)	C30—C35—C36—O9	−177.93 (13)
C16—C17—C18—N1	−178.34 (11)	C34—C35—C36—N3	179.69 (12)
C12—C17—C18—N1	4.60 (12)	C30—C35—C36—N3	0.88 (13)
C27—N2—C19—C3	113.18 (11)		

### Hydrogen-bond geometry (Å, º)

**Table:**

*D*—H···*A*	*D*—H	H···*A*	*D*···*A*	*D*—H···*A*
C7—H7*A*···O8^i^	0.98	2.47	3.2068 (15)	132
C8—H8*B*···O5^i^	0.98	2.34	3.2920 (15)	165
C9—H9*A*···O4^ii^	0.98	2.56	3.4742 (15)	156
C9—H9*C*···O9	0.98	2.38	3.2684 (19)	151
C10—H10*A*···O4	0.99	2.54	2.9144 (14)	102
C10—H10*B*···O3	0.99	2.32	2.8017 (15)	109
C19—H19*A*···O7	0.99	2.54	2.9200 (15)	103
C19—H19*B*···O2	0.99	2.36	2.8457 (14)	109
C23—H23···O1^iii^	0.95	2.45	3.3231 (16)	153
C28—H28*A*···O8	0.99	2.52	2.9187 (16)	103
C28—H28*B*···O3	0.99	2.34	2.8289 (16)	109
C32—H32···O2^iv^	0.95	2.56	3.2917 (16)	134
C34—H34···O7^v^	0.95	2.46	3.4066 (15)	172

Symmetry codes: (i) −*x*, −*y*+1, −*z*+1; (ii) −*x*, −*y*+1, −*z*; (iii) −*x*+1, −*y*+1, −*z*; (iv) −*x*+1, −*y*, −*z*+1; (v) *x*, *y*−1, *z*.
